# Targeting of vasoactive intestinal peptide receptor 2, VPAC2, a secretin family G-protein coupled receptor, to primary cilia

**DOI:** 10.1242/bio.201410561

**Published:** 2014-11-04

*Biol. Open* 2013, 2:686–694.

In this paper, GPCRs were screened for their ability to target to primary cilia. VPAC2 was reported as a novel ciliary targeting GPCR. Furthermore, the ciliary targeting signal of VPAC2 was identified and its ciliary targeting mechanisms were studied.

A departmental investigation into a previously published paper in a different journal resulted in a retraction stating that the first author, Livana Soetedjo, “had manipulated some of the figures”. The investigative committee recommended that the authors re-examine and re-test aspects of their *Biology Open* paper. The authors contacted the *Biology Open* Editorial Office when they were unable to repeat the results. Investigations further revealed that the first author “manipulated and fabricated data”, and they were unable to repeat the finding that VPAC2-GFP targets to cilia. Thus, the authors regret that they must retract their paper.

They apologize to the editors, reviewers and the scientific community for any loss of time or resources caused by this publication.

The journal was unable to make contact with the first author.

Hua Jin and De'Vona A. Glover

